# Predicting Alloreactivity in Transplantation

**DOI:** 10.1155/2014/159479

**Published:** 2014-04-28

**Authors:** Kirsten Geneugelijk, Kirsten Anne Thus, Eric Spierings

**Affiliations:** Laboratory for Translational Immunology, UMC Utrecht, Heidelberglaan 100, 3584 CX Utrecht, The Netherlands

## Abstract

Human leukocyte Antigen (HLA) mismatching leads to severe complications after solid-organ transplantation and hematopoietic stem-cell transplantation. The alloreactive responses underlying the posttransplantation complications include both direct recognition of allogeneic HLA by HLA-specific alloantibodies and T cells and indirect T-cell recognition. However, the immunogenicity of HLA mismatches is highly variable; some HLA mismatches lead to severe clinical B-cell- and T-cell-mediated alloreactivity, whereas others are well tolerated. Definition of the permissibility of HLA mismatches prior to transplantation allows selection of donor-recipient combinations that will have a reduced chance to develop deleterious host-versus-graft responses after solid-organ transplantation and graft-versus-host responses after hematopoietic stem-cell transplantation. Therefore, several methods have been developed to predict permissible HLA-mismatch combinations. In this review we aim to give a comprehensive overview about the current knowledge regarding HLA-directed alloreactivity and several developed *in vitro* and *in silico* tools that aim to predict direct and indirect alloreactivity.

## 1. Introduction

Human leukocyte antigen (HLA) matching significantly reduces the risk of graft rejection and graft failure after solid-organ transplantation [1–3] and graft-versus-host disease (GvHD) after hematopoietic stem-cell transplantation (HSCT) [4–9]. These pathological conditions evolve due to an alloreactive immune response that is initiated through interaction of allogeneic HLA with antibodies or the T-cell receptor (TCR). The subsequent immune response directed against allogeneic HLA impairs transplant outcome, emphasizing the need to avoid alloreactive responses after transplantation.

The highly polymorphic HLA system can be subdivided into two major classical classes: HLA class I and HLA class II. In general, HLA class-I molecules (HLA-A, -B, and -C) present endogenous peptides of 8–11 amino acids in length that can be recognized by CD8+ T cells, while HLA class-II molecules (HLA-DR, -DQ, and -DP) present exogenous peptides of 13–18 amino acids in length that can be recognized by CD4+ T cells. HLA class-I molecules consist of a polymorphic alpha chain and a nonpolymorphic beta-2-microglobulin and have a rather closed peptide binding groove. On the other hand, HLA class-II molecules consist of a polymorphic alpha and beta chain and have a more open structure.

Acquiring HLA-matched donors for transplantation is very challenging, due to the high level of polymorphisms in the HLA system. HLA incompatible transplantations can therefore not be avoided for a large number of patients. In those cases where a fully HLA-matched donor is not available, there is a clinical need to predict whether a certain HLA mismatch will elicit severe B-cell and T-cell-mediated alloreactive responses or not. There is cumulating evidence that these high-risk HLA mismatches (so-called nonpermissible mismatches/unacceptable mismatches) and well-tolerated HLA mismatches (so-called permissible mismatches/acceptable mismatches) exist, as epidemiological studies have shown that permissibility of HLA-mismatched combinations is highly variable [6, 7, 10]. For example, HLA-B∗44:02 and HLA-B∗44:03 mismatching leads to the induction of allospecific CD8+ T cells* in vitro* [11] and bone marrow-allograft rejection* in vivo* [12]. The amino-acid sequences of HLA-B∗44:02 and HLA-B∗44:03 differ only in one amino acid [13], indicating that even small amino-acid changes between HLA molecules can result in major alloreactive immune responses after transplantation. On the other hand, HLA class-I mismatches that are highly diverse may well be tolerated in HSCT [14]. Differences in permissibility between HLA-mismatched combinations may be explained by a different impact of amino-acid polymorphisms on peptide-binding features. Some amino-acid sequence polymorphisms will alter peptide-binding motifs and peptide-HLA complex conformation, thereby potentially inducing alloreactive immune responses, while others will not alter peptide-HLA landscapes.

Characterizing the permissibility of HLA mismatches prior to transplantation allows selection of the most optimal donor-recipient match and thereby will help to diminish the risk of posttransplantation complications after HLA incompatible transplantations. However, epidemiological studies do not provide a universal tool for defining permissibility for every HLA-mismatched combination, as these data are limited to the specific HLA-mismatched combinations studied; very large study populations would be required to study all potential combinations. Several approaches have therefore been developed to define permissibility of HLA-mismatched combinations; some of these approaches are very useful in predicting alloreactivity. We here review the current knowledge regarding HLA-directed alloreactivity and the various* in vitro* and* in silico* methodsthat can be used to predict this alloreactivity.

## 2. Pathways of Allorecognition

HLA alloreactivity in transplantation involves both B-cell- and T-cell-mediated responses. Three mechanisms of alloreactivity directed towards allogeneic HLA have been described: direct, indirect, and semidirect allorecognition.

IgG HLA alloantibodies directly recognize intact allogeneic HLA molecules that are present on the cell surface. These antibodies play a pivotal role in solid-organ transplantation and probably also have a role in HSCT [15–18]. The humoral response directed against allogeneic HLA can be established upon exposure to allogeneic HLA during pregnancies, blood transfusions, or (previous) transplantations. During this response, allogeneic HLA antigens are internalized by B cells and processed into peptides. These peptides can subsequently be presented on HLA class-II molecules that are present on the cell surface. Recognition of these HLA class-II presented HLA-derived epitopes by CD4+ T cells results in B-cell activation and IgM to IgG isotype switching [19]. Donor-specific IgG HLA antibodies (DSA) that are subsequently produced can bind directly to small polymorphic amino-acid residue patches that are present on the molecular surface of HLA antigens [20–22], thereby inducing rejection of graft tissue/cells, designated as antibody-mediated rejection.

In addition to alloantibodies, alloreactive T cells can also directly recognize intact allogeneic HLA molecules [23]. There is compelling evidence that cross-reactive T cells are involved in direct T-cell recognition [24–31]. These cross-reactive T cells initially react towards a foreign peptide, for instance, a viral peptide, presented by self-HLA. However, these T cells can also respond to allogeneic HLA presenting a self- or viral peptide [24–31]. Although these cross-reactive T cells can persist over time, direct T-cell recognition is predominantly involved in the acute stage of alloreactivity [32]. Intact HLA molecules present on resident donor-derived antigen-presenting dendritic cells are considered to be the driving force behind direct recognition in solid-organ transplantation, since parenchymal cells within transplanted tissues are unable to induce direct T-cell recognition (reviewed in [33]). Because these dendritic cells are depleted over time, the contribution of direct recognition in chronic graft rejection after solid-organ transplantation is limited [32, 33].

In contrast to direct T-cell recognition, indirect T-cell recognition is considered to be mainly involved in later stages of alloreactivity [34]. During indirect recognition, T cells recognize processed epitopes derived from allogeneic HLA that are presented by HLA molecules that are likely shared between donor and recipient [35, 36], as T cells are restricted to self-HLA. Indirect T-cell recognition is also involved in the formation of HLA alloantibodies, since T-cell recognition of B-cell presented HLA epitopes is required in this process [19, 37]. Thus, indirect T-cell recognition may also partly contribute to early alloreactivity, as indirect recognition can amplify the direct recognition response.

In semidirect allorecognition, allogeneic HLA:peptide complexes are transferred from allogeneic cells to autologous dendritic cells, resulting in a chimeric antigen-presenting cell [38]. Transfer of allogeneic HLA:peptide complexes can be achieved through secretion of endosomes containing HLA:peptide complexes [39] or through cell-to-cell contact between donor and recipient dendritic cells [40]. Antigen-presenting cells that acquire intact allogeneic HLA:peptide complexes on their cell surface may elicit both direct and indirect alloreactive T-cell responses. Although* in vivo* evidence for the role of the semidirect allorecognition pathway in graft rejection and GvHD is limited, it has been shown that this pathway is able to elicit cytotoxic alloimmunity* in vitro* and* in vivo* [41] and that the transfer of allogeneic HLA:peptide complexes likely occurs in an* in vitro* system of GvHD [42]. These observations suggest that that semidirect allorecognition may be involved in posttransplantation complications.

## 3. Prediction of Direct HLA Recognition by Antibodies

Humoral sensitization to HLA class-I and class-II epitopes and the subsequent production of HLA-specific antibodies can occur upon pretransplant exposure to allogeneic HLA. The presence of DSA before transplantation is related to antibody-mediated rejection and significantly impairs graft prognosis [15, 16]. Therefore, evaluation of HLA-sensitizing events (i.e., pregnancies, blood transfusions, and previous transplants) is generally included in standard pretransplantation screening. Pregnancy is a major contributor to HLA sensitization, as approximately 30% of the pregnancies results in child-specific sensitization towards HLA-A, -B, -C, and/or -DR loci [43]. Moreover, the HLA sensitization frequency increases with the number of full-term pregnancies [43]. Blood transfusions can induce HLA sensitization in approximately a third of the solid-organ transplantation recipients [44]. However, blood transfusions have a less prominent effect on HLA alloimmunization than pregnancy and solid-organ transplantation [44, 45]. In addition to the classical sensitizing events, HLA alloantibodies can also be raised against epitopes in allergens, ingested proteins, and microorganisms that are cross-reacting with HLA [46]. Although the presence of these “natural” DSA in kidney recipients is associated with the induction of mild episodes of antibody-mediated rejection, these patients have favorable graft outcome [47]. Therefore, the existence of these “natural” DSA prior to transplantation is currently not a contraindication for transplantation [47].

Although DSA detection methods are important tools for risk assessment prior to transplantation, pretransplant evaluation of preformed DSA remains challenging. For example, antibodies might become undetectable at the moment of transplantation due to the decay of antibody levels over time [48]. The clinical relevance of these preexisting low DSA levels is highly variable; some preformed DSA will elicit HLA alloreactivity* in vivo*, whereas others will not. Currently used detection methods may thus not detect the whole repertoire of clinically relevant DSA. Moreover, risk assessment of high DSA levels is also complicated. The complement-dependent cytotoxicity (CDC) crossmatch assay (reviewed in [49]) is a potent manner to measure the presence of clinically relevant antibodies, whereas other DSA detection assays, like the HLA-based enzyme-linked immunoabsorbent assay (ELISA) and Luminex-based assays [50, 51], provide valuable but limited information about the clinical relevance of identified DSA. Currently, the CDC assay seems to be a potent indicator for alloreactivity, while* in vitro* DSA detection methods can further support the matching procedure for solid-organ transplantation. Combining* in vitro* assays with an* in silico* prediction method allows identification of acceptable HLA mismatches towards which a recipient will likely not develop antibody-mediated responses.

### 3.1. *In Vitro* DSA Screening Assays

Assessment of humoral sensitization to allogeneic HLA was initially performed by the CDC crossmatch assay [49]. This assay measures the presence of preformed or* de novo *formed antibodies through their induction of complement-dependent lymphocyte killing. A positive CDC test was associated with a significantly impaired outcome after kidney transplantation [49, 52]. Despite its potency to mimic the* in vivo* situation, CDC crossmatch assays lack sensitivity [49] and may show false positive results [53]. To overcome these problems, a more sensitive assay was developed: the flow cytometry-based crossmatch (FCXM) assay [54]. However, both FCXM and the classical CDC crossmatch test correlate equally well to clinical outcome after kidney transplantation [55].

The lack of sensitivity and specificity of cytotoxicity crossmatch assays has led to development of solid-phase assays, such as the HLA-based ELISA and Luminex assays [50, 51]. These solid-phase methods, particularly Luminex, are very sensitive and specific; relevant anti-HLA class-I and class-II antibody profiles in solid-organ transplant recipients can be identified and monitored over time. Combining antibody profiles that are present in solid-organ transplant recipients, with HLA typing of the donor, designated as virtual crossmatching, allows identification of DSA and therefore might be useful in risk stratification prior to solid-organ transplantation (reviewed in [56, 57]). Unfortunately, estimation of the clinical relevance of DSA detected with solid-phase assays remains challenging [56], as tools to discriminate between nondetrimental DSA and deleterious DSA are lacking. Nevertheless, the presence of class-I and class-II DSA detected by Luminex in the absence of positive CDC assay is suggested to be indicative for impaired graft outcome in kidney transplantation [58].

### 3.2. HLAMatchmaker

*In vitro* CDC-based DSA detection assays have their limitations; these assays are not suitable to determine HLA-mismatch permissibility for highly sensitized transplantation candidates. Because of the high sensitization levels in those individuals, CDC assays often become almost completely positive, which complicates selection of suitable CDC-negative donors that will not elicit HLA alloreactivity* in vivo*. Therefore, alternative* in silico* methods were sought to predict acceptability of HLA-mismatched combinations.

An established and well-accepted* in silico* method is HLAMatchmaker. The* in silico* algorithm HLAMatchmaker is based on the principle that HLA-specific alloantibodies can bind to distinct amino-acid polymorphisms (immunogenic epitopes) present on HLA antigens [20, 21]. Multiple polymorphic amino-acid residues on the molecular surface of HLA antigens have been identified. Some of these residues are inaccessible for antibodies, since they are located near the cell membrane or within peptide-binding groove of the HLA molecule, while other residues are fully accessible for antibodies [20, 21]. HLAMatchmaker uses this knowledge to predict which HLA mismatches are not able to induce complications in transplantation recipients by defining the acceptable mismatches [20, 21]. Initially, HLAMatchmaker defined immunogenic epitopes as antibody-accessible, linear sequences of amino-acid polymorphisms (triplets) [20, 21]. Triplets that are present in donor HLA antigens, but not in the recipient HLA antigens, were considered to elicit humoral responses [20, 21]. On the other hand, triplets that are present in both donor and recipient HLA antigens were considered as acceptable [20, 21]. Thus, HLAMatchmaker provides a tool for identification of acceptable HLA mismatches.

The clinical applicability of HLAMatchmaker in matching strategies has been extensively evaluated. It has been shown that the triplet version of HLAMatchmaker is a potent indicator for the presence and magnitude of allogeneic HLA-directed antibody responses in renal transplantation and during pregnancy [59, 60]. In contrast, the number of triplet mismatches was not indicative for the induction of T-cell alloreactivity [61]. This lack of correlation between triplets and T-cell alloreactivity is probably caused by alternative epitope binding by T cells or by the involvement of larger polymorphic sequences in T-cell alloreactivity [61]. With regard to HSCT, the number of triplets did not correlate to acute GvHD, engraftment, or survival [62].

Despite its applicability in HLA-matching strategies, the triplet version of HLAMatchmaker represents an incomplete repertoire of immunogenic epitopes, as only linear sequence positions are implemented [22]. This hiatus has resulted in the development of a redefined version of HLAMatchmaker that identifies eplets [22]. Eplets are immunogenic HLA epitopes that are critical for antibody binding and consist of polymorphic amino-acid patches located at the molecular surface of HLA molecules [22]. These polymorphic patches may consist of polymorphisms in linear sequence positions and three-dimensional polymorphic patches in discontinuous sequence positions. Therefore, implementation of eplets into the algorithm has led to a more accurate definition of structural HLA epitopes.

Evaluation of the eplet version of HLAMatchmaker has shown a similar performance in predicting allogeneic HLA acceptability compared to the triplet version [60]. Nevertheless, the eplet version of HLAMatchmaker provides further discrimination of highly divergent HLA specificities [60]. Although conflicting results were reported with regard to the prognostic information that is provided by HLAMatchmaker on graft outcome ([63–66], reviewed in [67]), it is generally accepted that HLAMatchmaker is a suitable tool to analyze serum antibodies and to identify acceptable mismatches in solid-organ transplantation [67]. However, HLAMatchmaker is inappropriate for HSCT donor selection [62].

In addition to the number of eplets as determined by HLAMatchmaker, additional determinants can be used to define allogeneic HLA acceptability, for instance, physiochemical properties of polymorphic amino acids [68]. Differences in physiochemical properties between mismatches, including electrostatic potential and hydrophobicity, are useful to predict HLA class-I- and class-II-specific alloantibody responses prior to solid-organ transplantation [68–70]. With higher physiochemical disparity between HLA mismatches, the risk of antibody development increases after kidney transplantation [68–70]. These observations suggest that differences in physiochemical properties between polymorphic amino acids may be relevant in defining acceptable HLA mismatches. However, evidence to support clinical relevance is currently lacking.

## 4. Prediction of Direct T-Cell Recognition

The presence of T-cells directly recognizing intact allogeneic HLA molecules was previously shown in individuals suffering from graft rejection after solid-organ transplantation [71, 72] and GvHD after HSCT [73]. There is compelling evidence that direct T-cell alloreactivity results from cross-reactive T cells that are initially primed by a foreign peptide, for instance, a viral peptide [24–31]. For example, the HLA-B∗08:01-presented EBV peptide FLRGRAYGL is recognized by an EBV-specific TCR [74], that possesses cross-reactive capacities towards HLA-B∗44:02-presented peptide EEYQAFTY [24, 75]. Thus, the HLA-B∗08:01-presented peptide FLRGRAYGL elicits a public immune response. During a public immune response, the immune response directed against an identical epitope is dominated by T cells expressing similar TCRs in multiple subjects [76]. Since virus-specific T cells can be detected in high levels in healthy individuals [77], it is likely that cross-reactive virus-specific T cells may be present in both solid-organ transplantation recipients and HSCT donors prior to transplantation. The presence of virus-specific T cells that are cross-reactive with allogeneic HLA in these individuals may significantly contribute to complications after transplantation. However, most virus-specific T-cell responses do not have the propensity to induce public TCR responses nor predictable cross-reactivity with allogeneic HLA [78]. A single viral infection can therefore result in the establishment of multiple T cells that are cross-reactive to multiple HLA molecules, whereas other viral infections do not give rise to these cross-reactive T cells. In addition, virus-specific T cells with the same antigen specificity, but different TCRs, elicit different unpredictable patterns of alloreactivity [26, 79].

The molecular mechanism behind T-cell cross-reactivity is complex and currently incompletely understood. T-cell cross-reactivity assumably arises due to structural homology of HLA:peptide complexes (reviewed in [78]) rather than sequence homology of the presented peptides. Despite their sequence dissimilarity, the structure of FLRGRAYGL and EEYQAFTY epitopes in the context of their presenting HLA molecules is quite similar [75]. Therefore, molecular mimicry likely attributes to the observed cross-reactivity between these epitopes. On the other hand, cross-reactive TCR in mice can dock to self-MHC:peptide complexes in a different orientation than to allogeneic MHC:peptide complexes, suggesting that cross-reactivity can be established without molecular mimicry [80]. Thus, direct alloreactivity is a complex immune response that can only partially be explained by molecular mimicry. Since the molecular mechanism behind direct T-cell recognition is poorly understood, prediction of alloreactivity based on viral history is complex. Knowledge about viral history is therefore not sufficient to predict direct T-cell alloreactivity directed towards allogeneic HLA. Since direct T-cell allorecognition was studied intensively over the past decades, several alternative approaches to predict direct T-cell alloreactivity* in vitro* and* in silico* have been developed.

### 4.1. Cytotoxic T Lymphocyte Precursor Assays

Cytotoxic T lymphocyte precursor assays (CTLp) determine permissibility of HLA mismatches through* in vitro* evaluation of effector cytotoxic T-cell induction. This chromium 51 (^51^Cr) release-based assay, initially described by Brunner et al. [81], estimates cytotoxic T-cell activity directed against allogeneic HLA [82]. Further development of this assay has resulted in an assay that estimates the extent of alloreactive T-cell responses directed towards allogeneic HLA [82]. Since individual allogeneic HLAs can be linked to CTLp frequencies, these assays are a useful approach to distinguish between permissible and nonpermissible mismatches* in vitro* [83]. More importantly, a high CTLp frequency correlates reasonably well with clinical outcome* in vivo*; high CTLp frequencies were associated with graft rejection after solid-organ and tissue transplantation [73, 84, 85] and with GvHD and impaired survival after allogeneic HSCT [82, 83, 86]. Association between graft failure and the presence of primed cytotoxic T lymphocytes in sensitized transplant candidates (e.g., women after previous pregnancy) was shown as well [87].

Despite the usefulness of CTLp assay in estimating T-cell alloreactivity, the time-consuming and laborious character of this assay is a major drawback [82]. In order to overcome these disadvantages, alternative* in silico* approaches have been sought that mimic the CTLp assay result [88]. To this end, amino-acid polymorphisms at TCR-recognition and peptide-binding regions between HLA class-I mismatches were analyzed for their physiochemical and/or position characteristics and were correlated to CTLp outcome [88]. These analyses resulted in the establishment of a novel algorithm, which aims to predict HLA class-I mismatch-specific CTL alloreactivity [88]. Although the algorithm can predict CTLp outcome reasonably well, usage of this model for donor selection seems limited; this tool does not predict GvHD development in patients receiving HSCT [89].

### 4.2. T-Cell Epitope Model

The first clinically relevant model that successfully estimates the effect of direct recognition in HSCT has recently been developed. This HLA-DPB1-restricted model is designated as the T-cell epitope (TCE) model [90]. This model has been based on* in vitro* data from two alloreactive T-cell clones isolated from an HSCT patient with graft rejection due to an HLA-DPB1 mismatched graft [90]. Membrane-bound intact HLA was essential for recognition of the HLA-DPB1 mismatch by the alloreactive T-cell clones; the clones did not respond to B-lymphoblastoid cell lines transduced with a truncated mismatched-HLA-DPB1 construct that did not lead to cell-surface expression of HLA-DPB1 [90]. Thus, it seems likely that these two alloreactive T-cell clones recognized the HLA-DPB1 mismatched antigen in a direct manner.

In order to identify patterns of recognition of other alleles, the T-cell clones were further tested for their recognition of other HLA-DPB1 alleles [90]. Alleles were divided into three different immunogenic levels: highly immunogenic (i.e., both clones recognized the alleles), intermediate immunogenic (i.e., one of the clones recognized the allele but the other did not), or nonimmunogenic (i.e., both clones did not recognize the allele). Since testing of all HLA-DPB1 alleles* in vitro* is very time consuming, immunogenicity of other HLA-DPB1 alleles was extrapolated, based on similarities between the peptide-binding grooves of the* in vitro* tested alleles and the not-tested HLA-DPB1 alleles.

Subsequently, HLA-DPB1 mismatches were labeled as permissive or nonpermissive based on their immunogenic level and the concept of thymic education. For example, when the HLA-DPB1 allele of the donor belongs to the highly immunogenic group, then donor T cells should be educated not to respond to HLA-DPB1 alleles belonging to the highly immunogenic group and, in theory, will also not respond to lower immunogenic alleles. Therefore, when the recipient has an HLA-DPB1 allele belonging to the same or a lower immunogenic group, then the HLA-DPB1 mismatch will be permissive in the graft-versus-host (GvH) direction. On the other hand, since the HLA-DPB1 allele of the recipient is not immunogenic, recipient T cells are able to respond to (higher) immunogenic alleles of the donor. Thus, such mismatches are nonpermissive in the host-versus-graft (HvG) direction.

Nonpermissive mismatches, defined by the TCE model, are highly correlated to alloreactivity as reflected by GvHD, graft rejection, and transplant-related mortality after HLA-DPB1-mismatched HSCT [4, 90–92]. Counterintuitively, in these situations, the direction of the nonpermissiveness appears not to be important: both HvG and GvH nonpermissive mismatches lead to alloreactivity in the GvH direction (i.e., GvHD) [4, 91]. Therefore, both HvG and GvH nonpermissive mismatches are considered overall nonpermissive [4, 91]. The underlying biology of this bidirectional nonpermissiveness is currently poorly understood.

### 4.3. HistoCheck

The* in silico* model HistoCheck has been developed to estimate T-cell alloreactivity between HLA class-I and class-II mismatches [93]. HistoCheck calculates a matching score for any donor-recipient combination based on their HLA typing, the so-called sequence-similarity matching score [93]. The sequence-similarity matching score is determined by comparing differences in amino acids between HLA alleles with regard to their functional similarity and their location in the HLA molecule; amino-acid positions involved in TCR recognition and HLA-peptide binding are implemented in the sequence-similarity matching score [93]. As a high sequence-similarity matching score represents a high level of dissimilarity between donor and recipient [93], correlation of the sequence-similarity matching scores with clinical outcome was expected. However, HistoCheck is not indicative for transplant outcome* in vivo*, as sequence-similarity matching scores showed no correlation with GvHD after HSCT [94–96]. The inability of HistoCheck to be indicative for T-cell alloreactivity may be explained by several limitations of this model: HistoCheck does not integrate the presence of alloreactive donor T cells nor viral history in its algorithm. Additionally, the concepts of aforementioned molecular mimicry between HLA:peptide complexes and unconventional docking of TCR are not included in HistoCheck. Since these aspects of direct T-cell recognition are complex and not fully understood, establishment of reliable, clinically relevant tools to predict direct T-cell recognition remains challenging.

### 4.4. Prediction Based on Specific Amino-Acid Changes

An alternative approach to predict direct T-cell alloreactivity is to analyze the impact of amino acids at certain locations within HLA molecules. Several amino-acid substitutions in the peptide-binding domain of HLA class-I molecules are related to an increased risk of GvHD [6], whereas other amino-acid substitutions are related to a diminished relapse risk [7]. The effect of specific amino-acid changes on alloreactivity was recently investigated in a large cohort [97]. In this study, the impact of changes on HLA class-I positions 9, 99, 116, and 156 for peptide binding alteration and position 77 for killer cell immunoglobulin-like receptor binding was investigated in recipients of an allogeneic HSCT with a single allelic mismatch at either the HLA-A, -B, or -C locus [97]. Particularly amino-acid changes at position 116 in HLA-C were associated with an increased acute GvHD risk [97, 98], but also changes at position 99 for HLA-C and position 9 for HLA-B were associated with clinical T-cell alloreactivity [97]. By determining the effect of specific amino acids within the HLA molecule, multiple amino-acid positions have been identified that influence transplantation outcome; this knowledge may be used for donor selection.

## 5. Prediction of Indirect T-Cell Recognition

Indirect recognition of allogeneic HLA acts via presentation of peptides derived from allogeneic HLA molecules. Over 350 of these indirectly recognizable HLA-derived peptides have been eluted from HLA [99]. T-cells recognizing these peptides likely play a role in alloreactivity; the erection of indirectly recognizing T cells after solid-organ transplantation was strongly correlated to both acute [100–102] and chronic graft failure [102, 103]. Furthermore, the presence of circulating T-cells recognizing allogeneic HLA epitopes in an indirect manner was predictive of rejection [101]. As mentioned previously, indirect T-cell recognition is considered to be a slower alloreactive response than direct T-cell recognition [33, 34]. The proposed slower rate of indirect T-cell recognition may be related to the idea that indirectly recognizing T cells arise from the naive pool, whereas directly recognizing T cells likely evolve from the memory pool, as the latter T cells are supposedly cross-reactive [104]. Since direct recognition has received most attention historically, not many methods are available to predict indirect recognition of HLA disparities; there is no* in vitro* system available and only one* in silico* model.

### 5.1. PIRCHES Model

We have recently developed a model for* in silico* prediction of indirectly recognizable HLA-derived peptides, the so-called PIRCHES model (predicted indirectly recognizable HLA epitopes) [105]. Indirect T-cell recognition that targets allogeneic HLA depends on HLA-derived peptides that differ between host and graft. These HLA-derived peptides are likely presented on shared HLA. HLA-derived peptides that are identical between donor and recipient should be ignored by the alloimmune system, as T-cells recognizing these peptides should have been deleted from the repertoire due to thymic selection. Thus, the HvG reaction of graft rejection after solid-organ transplantation should be evoked by donor-specific peptides, whereas GvHD after HSCT should be evoked by recipient-specific peptides. We have designated the donor-specific peptides that can be recognized by the recipient as HvG-PIRCHES and the recipient-specific peptides that can be recognized by the donor as GvH-PIRCHES.

In order to elicit indirect T-cell recognition, allogeneic HLA proteins need to be processed into peptides and these peptides need to be presented on shared HLA. Since both steps are determined by certain motifs in the protein sequences, both antigen processing and antigen presentation pathways can be predicted via several (computational) tools [106–120]. Our PIRCHES model uses these predictions to define permissibility of HLA mismatches. For HLA class-I peptide presentation (designated as PIRCHE-I), the PIRCHES model first determines proteasomal cleavage of all HLA molecules of the donor and recipient into peptides and transport of those peptides via the transporter associated proteins (TAP) into the endoplasmic reticulum (ER). Subsequently, the binding affinities of the predicted cleavage products to HLA class-I alleles are predicted, as a derivative of peptide presentation by the HLA class-I molecules that are shared between donor and recipient. Prediction of HLA class-II-presented epitopes (PIRCHE-II) is restricted to HLA-binding affinity predictions of peptides, since (enzymatic) cleavage patterns have not been clearly defined yet.

On the basis of their performance, we implemented NetChop, NetMHCPan, and NetMHC-II or NetMHCIIPan (reviewed in [106–109]) in our PIRCHES model to predict the number of PIRCHE-I and PIRCHE-II. NetChop is a potent predictor of proteasomal cleavage and TAP transport, whereas NetMHCPan predicts binding affinity to HLA class I. NetMHC-II can predict peptide binding to HLA class-II alleles for which binding data exist, whereas for NetMHCIIPan these data were extrapolated to other alleles. Both HLA class-I- and HLA class-II-binding predictors have good predictive capacities [106] and are frequently used to identify viral epitopes [121].

The first construction of the PIRCHES model was based on predicting HLA class-I-derived peptide presentation on shared HLA-DR and used the binding affinity predictions of NetMHC-II [105]. After kidney transplantation with HLA class-I mismatches, mismatches that led to allogeneic HLA-specific antibody production correlated to higher numbers of HvG-PIRCHE-II compared to mismatches that did not led to antibody production [105], suggesting that indirect recognition of HLA-derived epitopes was required for HLA-specific IgG antibody production. For HSCT, the situation is more difficult, as alloreactivity after HSCT not only involves CD4+ T-cell recognition and stimulation of B cells but clearly involves CD8+ T-cell recognition of alloantigens as well [122]. The PIRCHES model was therefore extended to PIRCHE-I predictions. Moreover, usage of NetMHCIIPan was incorporated, as NetMHC-II can only predict binding to a limited number of HLA-DR alleles. Indeed, after HLA-mismatched HSCT, high numbers of both GvH-PIRCHE-I and -II are correlated to clinical alloreactivity (Thus et al., manuscripts in preparation).

In the current PIRCHES model, we regarded any difference in presentable peptides derived from donor-versus-recipient alleles as a PIRCHE (i.e., only one amino-acid difference is regarded as difference). The model can likely be improved when the T-cell recognition is more specifically elucidated. It is well known that some positions of peptides are more important in TCR binding than others [123, 124], as amino acids that are lying deep inside the peptide binding groove of the presenting HLA molecule are likely not seen by the TCR. Furthermore, polymorphisms leading to different peptide properties (e.g., polar versus nonpolar and hydrophobic versus hydrophilic) may lead to more pronounced T-cell recognition. These refinements are currently being studied for their effect on the predictive potential of the model.

## 6. Conclusion

HLA mismatches can cause severe posttransplantation complications such as graft rejection [1] and GvHD [5]. In these complications, the induction of both antibody production and T-cell recognition may play a role. Interestingly, permissibility of HLA-mismatched combinations is highly variable; some mismatches are poorly tolerated, whereas others are highly permissible. Although the degree of HLA amino-acid sequence disparity varies largely amongst different HLA mismatches depending on the allelic versus antigenic nature of the mismatch and the HLA locus, the number of polymorphic amino-acid residues in itself is not predictive for the permissibility of HLA-mismatched combinations, as multiple additional factors are involved. Both the nature and the position of the amino-acid polymorphisms within the mismatched HLA, as well as their effect on neighboring amino acids, determine the permissibility of HLA-mismatched combinations. Several approaches have been developed to predict the permissibility of HLA mismatches, thereby aiming to improve donor selection procedures. The objective of all these approaches is to predict the development of the abovementioned antibody and T-cell recognition of allogeneic HLA.

Several well-established* in vitro* assays can be used to detect DSA that are related to impaired graft survival. In addition to these assays, HLAMatchmaker is a well-validated tool to identify which HLA mismatches do not induce alloreactive humoral responses in transplantation recipients [20]. Although HLAMatchmaker is a powerful predictor for acceptable HLA mismatches in solid-organ transplantation, this tool is not suitable for predicting HLA permissibility in the setting of HSCT [62].

With regard to direct T-cell recognition, the risk for clinical alloreactivity can be estimated with the* in vitro* CTLp assay [82]. In addition to this* in vitro* assay, several* in silico* approaches aim at predicting direct recognition-based T-cell alloreactivity. For example, the TCE model can assess nonpermissive HLA-DPB1 mismatches for HSCT [90]. The relevance of the TCE model has not yet been investigated in solid-organ transplantation. Practically, one should note that HLA-DPB1 is rarely typed prospectively in the setting of solid-organ transplantation, as donor availability is more restricted than for HSCT. Although HistoCheck has been developed to estimate direct recognition* in silico* for all HLA loci, this model does not correlate to alloreactivity* in vitro* nor* in vivo* [95]. Alternatively, several studies have identified amino-acid positions that are influencing transplantation outcome [97]; this information can be implemented in donor selection procedures.

Indirect T-cell recognition can be predicted with the* in silico* PIRCHES model [105]. This model predicts HLA-derived epitopes that can be presented on shared HLA classes I and II. Both PIRCHE-I and PIRCHE-II are well correlated to alloreactivity after HSCT. With regard to PIRCHE-II, increasing numbers of PIRCHE-II are correlated to antibody production after solid-organ transplantation [105].

Alloreactivity after transplantation can unlikely be attributed to one single pathway of HLA recognition. To determine the relative contribution of direct and indirect recognition, combining the different methods of predicting alloreactivity would be of interest. Direct and indirect recognition may act synergistically, and therefore the combination of a positive CTLp assay and a high number of PIRCHES may lead to a more pronounced alloreactive response. Furthermore, combining the PIRCHES and the TCE models for HLA-DPB1 mismatches might allow identification of HLA-DPB1 mismatches recognized in both direct and indirect manners. Moreover, a combination of low PIRCHE-II and low number of eplets as determined by HLAMatchmaker may be favorable in solid-organ transplantation.

In conclusion, over the past decades, many approaches have been developed to predict alloreactivity after transplantation* in vivo*, some attempts leading to more successful predictors than others. The failure of multiple tools to predict alloreactivity is not surprising, as knowledge about alloreactivity is still limited. However, multiple approaches seem to be clinically relevant and some are currently implemented in clinical practice. Further improvement of the definition of HLA-mismatch permissibility, and implementation of these definitions into the donor-selection procedure, will eventually lead to reduced alloreactivity, thereby improving clinical outcome after solid-organ transplantation and HSCT.
